# Segmentation and intensity estimation for microarray images with saturated pixels

**DOI:** 10.1186/1471-2105-12-462

**Published:** 2011-11-30

**Authors:** Yan Yang, Phillip Stafford, YoonJoo Kim

**Affiliations:** 1 School of Mathematical and Statistical Sciences, Arizona State University, Tempe, AZ 85287, USA; 2 Center for Innovations in Medicine, Biodesign Institute, Arizona State University, Tempe, AZ 85287, USA; 3 School of Mathematical and Statistical Sciences, Arizona State University, Tempe, AZ 85287, USA

## Abstract

**Background:**

Microarray image analysis processes scanned digital images of hybridized arrays to produce the input spot-level data for downstream analysis, so it can have a potentially large impact on those and subsequent analysis. Signal saturation is an optical effect that occurs when some pixel values for highly expressed genes or peptides exceed the upper detection threshold of the scanner software (2^16 ^- 1 = 65, 535 for 16-bit images). In practice, spots with a sizable number of saturated pixels are often flagged and discarded. Alternatively, the saturated values are used without adjustments for estimating spot intensities. The resulting expression data tend to be biased downwards and can distort high-level analysis that relies on these data. Hence, it is crucial to effectively correct for signal saturation.

**Results:**

We developed a flexible mixture model-based segmentation and spot intensity estimation procedure that accounts for saturated pixels by incorporating a censored component in the mixture model. As demonstrated with biological data and simulation, our method extends the dynamic range of expression data beyond the saturation threshold and is effective in correcting saturation-induced bias when the lost information is not tremendous. We further illustrate the impact of image processing on downstream classification, showing that the proposed method can increase diagnostic accuracy using data from a lymphoma cancer diagnosis study.

**Conclusions:**

The presented method adjusts for signal saturation at the segmentation stage that identifies a pixel as part of the foreground, background or other. The cluster membership of a pixel can be altered versus treating saturated values as truly observed. Thus, the resulting spot intensity estimates may be more accurate than those obtained from existing methods that correct for saturation based on already segmented data. As a model-based segmentation method, our procedure is able to identify inner holes, fuzzy edges and blank spots that are common in microarray images. The approach is independent of microarray platform and applicable to both single- and dual-channel microarrays.

## Background

Microarray technology has been used in many areas of biomedical research and drug development to study the function of thousands of genes in a single experiment. As an important early step in microarray studies, microarray image analysis produces the input spot intensity data to downstream analysis such as classification and identification of differentially regulated genes. Thus, image processing can have profound effects on those and subsequent analysis. Microarray images with saturated hybridization signals are common when the dynamic range of expression of biological quantities is large. To enhance weak signals, a common practice is to increase the photometric gain at scanning. A large gain, however, often causes some pixels for highly expressed genes to exceed the scanner's upper limit of detection. Discarding spots with saturated pixels can fail to detect target genes that are highly and differentially expressed, whereas using saturated values without correction tends to underestimate expression levels and distort high-level analysis [1].

Correcting saturation-induced bias for expression microarrays has triggered much of recent research. Early work involves combining spot intensity data from multiple scans, obtained at different laser power or photomultiplier tube (PMT) settings, into an extended linear range and estimating expression levels beyond the saturation threshold by extrapolation [2-4]. Wit and McClure [5] proposed a maximum likelihood (ML) approach in which censoring was incorporated to account for signal saturation using the mean, median and variance of each spot. Dodd *et al*. [6] developed a censored Gaussian regression model by exploiting the association between pixel intensities of two channels in dual-dye experiments. Along the line of saturation adjustments by censoring, Ekstrom *et al*. [7] considered parametric spot shape/profile models for pixel-level data and imputed the values for saturated pixels. A Cauchy distribution was employed in Khondoker *et al*. [8] to model spot intensity data with outlying observations from multiple scans. To account for saturation, the Cauchy location function was specified to follow the functional form of the mean of a censored Gaussian. Glasbey *et al*. [9] imputed censored pixel values based on the principal components of uncensored spots. Bayesian hierarchical modeling for handling signal saturation using data from multiple scans was considered by Gupta *et al*. [10] and Gupta *et al*. [11].

The use of spot-level data in most previous work is largely motivated by the ready access to such data through standard output files of image processing software. However, signal saturation occurs at individual pixels that form a spot. So using raw pixel values could potentially provide more effective bias adjustments. We base our analysis on pixel-level data in this article. Instead of accounting for saturated pixels in isolation from image segmentation, as has been done in previous work reviewed earlier, we propose to combine model-based segmentation with spot intensity estimation to correct for saturation at the segmentation stage. In mixture model-based clustering of pixels, pixel values are typically assumed to follow a finite mixture of parametric distributions such as Gaussian [12-15]. When a portion of pixel values are saturated, the distribution assumption should be modified to reflect this feature. Consequently, cluster memberships of the pixels may be altered. Thus, accounting for saturation during image segmentation has the potential to improve the accuracy of segmentation, which in turn would lead to more effective spot intensity estimation.

Yang *et al*. [16] and Li *et al*. [14] provided excellent reviews on methods for segmenting microarray images without saturation. As a histogram-based segmentation method, mixture model-based clustering of pixels has a few advantages. Not only can it accurately recover irregularly shaped spots, such as commonly seen donut-shaped spots [17], but it can also identify blank spots and spots with fuzzy edges. Fuzzy edges are a bigger concern for saturated spots, since the optical flare caused by extremely bright pixels often distorts the local background estimate. In this article, we propose a model-based image segmentation and spot intensity estimation procedure to correct for signal saturation at the pixel level. A censored Gaussian mixture model (GMM) with no more than three mixture components is developed, in which the number of components is selected based on information criteria. The expectation-maximization (EM) algorithm is carried out for model estimation and implemented in R. Before applying the proposed method, it is necessary to perform automatic gridding (i.e., locating the spots on an array) to provide the data for segmentation. Since the arrays in our data examples were spotted in an orange-crate packing pattern to increase spot density, a hexagonal grid was used. To facilitate high-throughput analysis, we provide Matlab code that extracts the pixel intensity values and the coordinates of the pixels belonging to each spot after automatic gridding is done. Through microarray examples and simulation, we demonstrate that our method extends the dynamic range of measured expression and is effective in correcting saturation-induced bias. We also illustrate the influence of saturation adjustments on modifying clustering results and the impact of image processing on downstream classification. Source code and data are available at http://math.la.asu.edu/~yy/cgmm.html.

The rest of this article is organized as follows. First, the censored GMM is introduced for segmenting microarray images with saturated pixels and estimating spot-level intensities. An EM algorithm is used to estimate the model. Next, we illustrate the proposed method with peptide microarray images from a human Valley Fever diagnosis study and a canine lymphoma diagnosis study, and compare our method with regular GMM-based segmentation and intensity estimation without saturation correction. We conclude with the main findings and comment on future research.

## Methods

Microarray image processing involves three main steps: 1) gridding or addressing that finds the exact location of each spot, 2) image segmentation that determines which pixels form the signal and which pixels form the background, and 3) intensity estimation that quantifies the expression level for each spot. The proposed censored GMM focuses on the second and third tasks to correct for signal saturation. The raw data are 16-bit grayscale images stored as TIFF files. The input data for our segmentation and intensity estimation procedure are pixel intensity values belonging to individual spots after automatic gridding is done. In the following we present the microarray studies that produced the images for the data examples, automatic gridding and pixel intensity extraction, and model-based segmentation and spot intensity estimation.

### Microarray data

We considered a microarray platform that is still in its infancy but has profound implications for health monitoring and pre-symptomatic disease detection. The immunosignaturing microarray is composed of 10,000 unique, random-sequence peptides that are printed in standard microarray format. The peptide probes detect antibody changes in the serum samples and correlate them with changes in health status. Microarray images were obtained from an experiment on Valley Fever (Coccidioidomycosis) diagnosis in humans and an experiment detecting T-cell and B-cell lymphoma in dogs. Both studies were conducted at the Biodesign Institute of the Arizona State University using the Immunosignaturing Arrays developed by the Center for Innovations in Medicine [18,19]. Human subjects were consented and de-identified according to IRB Protocol# 0905004024. In the Valley Fever diagnosis study, 60 patients with the disease and 30 healthy controls were examined. The lymphoma diagnosis study examined the serum samples from 21 dogs with either B-cell or T-cell lymphoma and 20 healthy dogs.

The microarrays were spotted in an orange-crate packing pattern using piezo-electric deposition of 10 pL of 1 mg/ml peptide in 20 uM Hepes buffer, 10 uM EDTA, 5 uM TCEP, pH 6.7 (Applied Microarrays, Tempe, AZ). In both experiments, the serum samples were diluted 1:500 in incubation buffer and allowed to incubate on the microarray slide. A secondary antibody pre-labeled with either Alexafluor 647 or Alexafluor 555 (Invitrogen, Carlsbad, CA) was added to the solution to detect the primary antibodies. The slides were washed, dried, and scanned at 645 nm or 550 nm (according to the dye) using an Agilent 'C' laser scanner (Agilent, Santa Clara, CA).

### Automatic gridding and pixel intensity extraction

**Figure 1:**
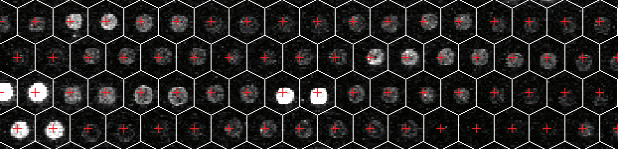
**Array image with a hexagonal grid superimposed**. Valley Fever diagnosis study: Array image with a hexagonal grid superimposed. The red +'s are spot centers.

Although the array layout parameters are known in advance, there are a number of sources that can lead to an imperfect grid. These include variations in print-tip positions and the spotting process, hybridization inconsistencies, and the need to produce highly dense arrays [20]. Slight departures from the pre-specified layout can result in considerable misalignments of the grid. To ensure the quality of image segmentation, one needs to first find the exact locations of the spots. The orange packing spotting pattern of the arrays requires the hexagonal grid instead of the more common rectangular grid. The boundaries of the hexagonal grid can be identified based on the spot centers, provided by the automatic spot finding algorithm of GenePix 6.0 [21] or other image processing software. We extract in Matlab the pixel intensity values and their coordinates within each target mask, the hexagon (or part of the hexagon for a spot on the edge) containing a spot and its surrounding background, and use them as the input data for model-based segmentation. Figure 1 shows the gridded image of a sub-block on an array from the Valley Fever diagnosis study. The spot locations are well identified.

### Model-based segmentation and intensity estimation

Let *Y_i _*denote the intensity value of pixel *i*, *i *= 1, ..., *n*, within a target mask on a digital image of one channel on a single-channel or double-channel microarray; *n *may vary for different spots. Assume that the *Yi*'s are independent and identically distributed draws from a *K*-component Gaussian mixture [22], in which the first *K *- 1 components are regular normal distributions, whereas the last component is a normal distribution right censored at the saturation threshold *S*. When the pixel intensities within a target mask do not roughly follow a mixture of Gaussian distributions, the data can be transformed to better conform with normality or other distributions may be assumed [8,15]. The mixture density for *y_i_*, a realized value of *Y_i_*, is then given by

$$\begin{array}{ll} f\left(y_{i};\theta \right)\; & =\overset{K-1}{\underset{k=1}{\sum }}\pi_{k}\phi \left(y_{i};\mu_{k},\sigma_{k}\right)\;\\ & \;+\pi_{K}\phi_{S}\left(y_{i};\mu_{K},\sigma_{K}\right),\;\\ & \; \end{array}$$

where 0 ≤ *π_k _*≤ 1, *k *= 1, ..., *K*, are the mixing weights that sum to one, *ϕ*(·;*μ_k_*, *σ_k_*) is the normal density function with mean *μ_k _*and standard deviation *σ_k_*, *ϕ_S_*(·; *μ_K_*, *σ_K_*) is the density function of a right-censored normal at *S *with mean *μ_K _*and standard deviation *σ_K_*, and ***θ ***= (*π*_1_, ..., *π*_*K *- 1_, *μ*_1_, ..., *μ_K_*, *σ*_1_, ..., *σ_K_*)*^T ^*is the vector containing all parameters. For parameter identification, let *μ*_1 _*< μ*_2 _< ... <*μ_K_*. For model-based clustering of pixels and spot intensity estimation, we maximize the log-likelihood function $l\left(\theta ;y\right)=\sum_{i=1}^{n}\log f\left(y_{i};\theta \right)$, where **y **= (*y*_1_, ..., *y_n_*)*^T^*.

**Figure 2:**
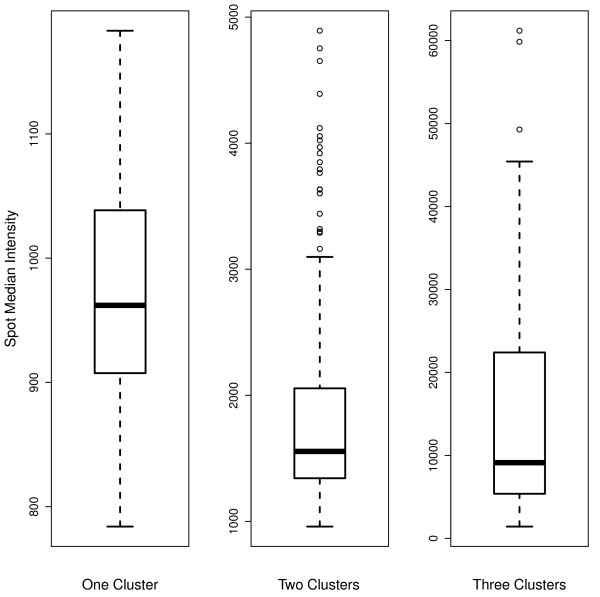
**Boxplots of foreground median intensities**. Valley Fever diagnosis study: Boxplots of foreground median intensities for blank spots, two-cluster spots and three-cluster spots on a random block with 484 spots. The number of clusters was selected by BIC.

The number of components *K *can be selected by likelihood-based information criteria (e.g., Bayesian information criterion or BIC). Similar to the work of Li *et al*. [14] for regular GMM-based clustering of pixels without saturation, we limit *K *to be a number no greater than three with the following arguments. When *K *equals 1, the target mask is associated with either a blank spot containing only background (BG) noise or a weak spot whose signal is too low to be differentiated from the BG. For *K *= 2, a "perfect" spot is resulted, in the sense that the two components correspond to the BG and the foreground (FG) signal, respectively. In practice, it may be common to observe *K *= 3; the extra component with an intermediate mean accounts for the fuzzy edge of a bright spot or the inner hole of a donut-shaped spot. Figure 2 depicts FG median intensities for a randomly selected block with 484 spots of an array image from the Valley Fever diagnosis study. Applying BIC to mixture component selection in our censored GMM-based segmentation, we identified 63 blank spots, 376 two-cluster spots, and 45 three-cluster spots. As shown in the figure, the three-cluster spots typically have much higher median intensities than the spots composed of only signal and BG. Thus, bright spots and saturated pixels further support the use of *K *= 3 in mixture model-based segmentation.

We next focus on the case of *K *= 3 to illustrate maximization of the likelihood *l*(***θ***; **y**) using the EM algorithm [23-25]. For *k *= 1 and 2, define the latent component indicator variables *Z_ik _*such that *Z_ik _*= 1 if *Y_i _*is from component *k *and *Z_ik _*= 0 otherwise. Then *Z*_*i*3 _= 1 - *Z*_*i*1 _- *Z*_*i*2_. Let *z*_*i*1_, *z*_*i*2 _and *z*_*i*3 _denote the realized values of *Z*_*i*1_, *Z*_*i*2 _and *Z*_*i*3_, and let (**y**, **z**) form the complete data, where **z**_1 _= (*z*_11_, ..., *z*_*n*1_)*^T^*, **z**_2 _= (*z*_12_, ..., *z*_*n*2_)*^T^*, **z**_3 _= (*z*_13_, ..., *z*_*n*3_)*^T^*, and $z={\left(z_{1}^{T},z_{2}^{T},z_{3}^{T}\right)}^{T}$. The log-likelihood function based on the complete data has the form

$$\begin{array}{ll} l\left(\theta ;y,z\right)\; & =l_{0}\left(\pi_{1},\pi_{2};z\right)+l_{1}\left(\mu_{1},\sigma_{1};y,z\right)\;\\ & \;+\;l_{2}\left(\mu_{2},\sigma_{2};y,z\right)+l_{3}\left(\mu_{3},\sigma_{3};y,z\right),\;\\ & \; \end{array}$$

where l0(π1,π2;z)= ∑i=1n(zi1 logπ1+zi2 logπ2+zi3 logπ3), l1(μ1,σ1;y,z)= ∑i=1nzi1 logϕ(yi;μ1,σ1), l2(μ2,σ2;y,z)= ∑i=1nzi2 logϕ(yi;μ2,σ2), and l3(μ3,σ3;y,z)= ∑i=1nzi3 log[I(yi<S)ϕ(yi;μ3,σ3)+I(yi=S){1-Φ(S-μ3σ3)}]; *I*(*A*) = 1 if event *A *occurs and equals 0 otherwise, and Φ(·) is the standard normal cumulative distribution function. It can be seen that the complete-data likelihood is decomposed into four parts: the log-likelihood *l*_0 _for a multinomial distribution on **z**, the weighted log-likelihoods *l*_1 _and *l*_2 _for two regular normal distributions on **y **with weights **z**_1 _and **z**_2_, and the weighted log-likelihood *l*_3 _for a right-censored normal on **y **with weight **z**_3_. The four likelihood parts, each with distinct parameters, can be maximized separately.

The EM is an iterative procedure for ML estimation that works with the complete data. Each iteration consists of an expectation (E) step and a maximization (M) step. Since the complete-data likelihood *l*(***θ***; **y**, **z**) is linear in the missing data **z**, the E step reduces to computing the conditional expectation of **Z **given the observed data **y **and parameter estimates from the previous iteration. The M step maximizes the four likelihood parts in *l*(***θ***; **y**, **z**) separately, each corresponding to a standard optimization problem. At iteration *t*, the conditional expectations of the *Z_ik_*'s are given by

$$\begin{array}{ll} z_{ik}^{\left(t\right)}\; & =\;E\left(Z_{ik}|y_{i},\theta^{\text{(}t-1\text{)}}\right)\;\\ & =\;\frac{\pi_{k}^{\left(t-1\right)}\phi \left(y_{i};\mu_{k}^{\left(t-1\right)},\sigma_{k}^{\left(t-1\right)}\right)}{f\left(y_{i};\theta^{\text{(}t-1\text{)}}\right)}\;\\ & \; \end{array}$$

for *k *= 1, 2 and $z_{i3}^{\left(t\right)}=1-z_{i1}^{\left(t\right)}-z_{i2}^{\left(t\right)}$. In the M step, the following closed-form solutions are obtained for *k *= 1, 2:

$$\begin{array}{ll} \pi_{k}^{\left(t\right)} & =\overset{n}{\underset{i=1}{\sum }}z_{ik}^{\left(t\right)}/n\;\\ \mu_{k}^{\left(t\right)} & =\overset{n}{\underset{i=1}{\sum }}z_{ik}^{\left(t\right)}y_{i}/z_{ik}^{\left(t\right)}\;\\ \sigma_{k}^{\left(t\right)} & =\overset{n}{\underset{i=1}{\sum }}z_{ik}^{\left(t\right)}{\left(y_{i}-\mu_{k}^{\left(t\right)}\right)}^{2}/\overset{n}{\underset{i=1}{\sum }}z_{ik}^{\left(t\right)}.\;\\ & \; \end{array}$$

For the censored normal component, estimators for *μ*_3 _and *σ*_3 _do not have analytical forms and need to be solved iteratively. The Newton-Raphson algorithm [26] for estimating a censored normal model iteratively evaluates the first and second derivatives of the likelihood component *l*_3_(*μ*_3_, *σ*_3_; **y**, **z**) with respect to the parameters. The method is implemented in standard software packages for survival analysis, such as the R survreg function in the survival library and the SAS LIFEREG procedure. Our R program implementation of the EM algorithm calls the survreg function in each M step to update the estimates of *μ*_3 _and *σ*_3_.

To start the iterative procedure, we obtain the initial values of the mean and scale parameters using the sample means and standard deviations calculated from appropriate portions of ordered pixel values within a target mask. The initial mixing weights are estimated based on prior knowledge about the typical spot and target mask sizes. In the Valley Fever diagnosis study, the initial value for *π*_1 _was taken as 0.8 in a two-component mixture and the values for *π*_1 _and *π*_2 _were 0.7 and 0.1 in a three-component mixture. Convergence of the algorithm is monitored by evaluating the increase in the likelihood function *l*(***θ***; **y**). At convergence, the ML estimate ${\hat{\mu }}_{1}$ of *μ*_1 _is used to quantify the BG noise for a spot. The spot signal is estimated by ${\hat{\mu }}_{K}$, given that *K *≥ 2. Consequently, the BG-corrected spot intensity is given by ${\hat{\mu }}_{K}-{\hat{\mu }}_{1}$ for *K *≥ 2. For a refined selection of *K*, one may specify a threshold for the relative difference in BIC below which a simpler model with a smaller *K*, even if it has a slightly larger BIC value, is preferred over a more complex model. A similar idea was used in Baek *et al*. [15] to flag spots with low expression levels for *K *≤ 2. The threshold value 0.001 for the relative difference in BIC was used in the data examples and the simulation study.

## Availability

Source code and sample data are made available at http://math.la.asu.edu/~yy/cgmm.html. Source code includes the R program for implementing the censored GMM-based segmentation and spot intensity estimation, and the Matlab program for extracting pixel intensity values and associated coordinates belonging to individual spots from an automatically gridded image, with either a rectangular grid or a hexagonal grid. Microarray images from the human Valley Fever diagnosis study are accessible through Gene Expression Omnibus Series accession number GSE33899 (http://www.ncbi.nlm.nih.gov/geo/query/acc.cgi?acc=GSE33899).

## Results and discussion

In this section we present the results from two data examples and a simulation study. We compare our method with the regular GMM similar to the segmentation method of Li *et al*. [14] and the fixed circle segmentation implemented in GenePix 6.0 [21]. Microarray images from a Valley Fever diagnosis study in humans were processed to illustrate the capability of the proposed method to enhance the dynamic range of expression data beyond the saturation threshold. A canine lymphoma diagnosis study was used to demonstrate the impact of saturation adjustments at the segmentation stage on downstream classification between healthy and cancer tissue. A simulation study was also conducted to evaluate the selection of *K *and the performance of the censored GMM in correcting saturation-induced bias.

### A human Valley Fever diagnosis study

Microarray images were obtained from a Valley Fever diagnosis study for identifying peptides that more effectively predicted the status of Valley Fever patients than a standard test. Each array consisted of 12 by 4 blocks and each block contained 22 by 22 spots, with 400-500 pixels per target mask. The hybridized arrays were scanned at 100% laser power and 70% PMT voltage, resulting in a few spots saturated at *S *= 65, 535.

**Figure 3:**
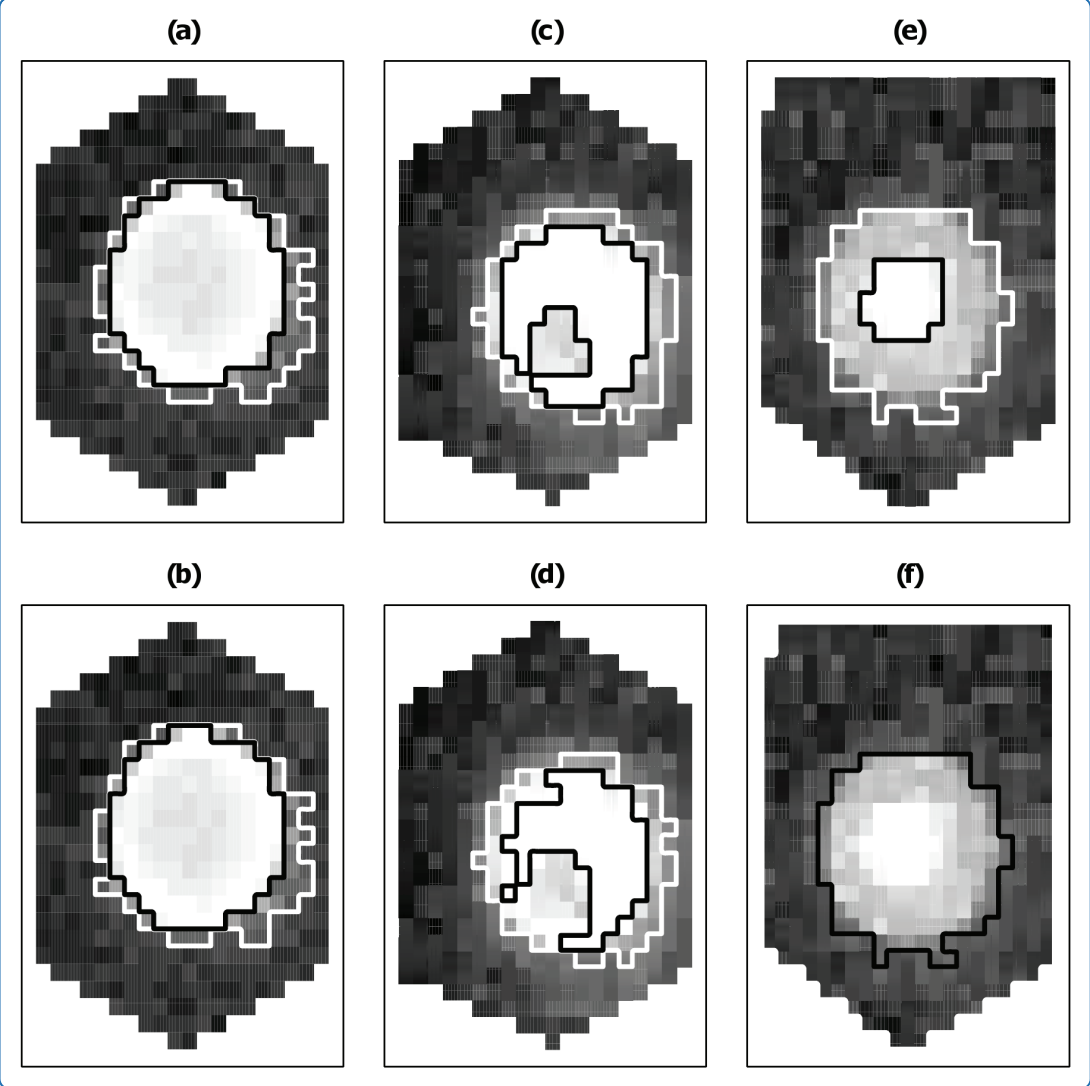
**Three saturated spots segmented by censored GMM and regular GMM**. Valley Fever diagnosis study: Three saturated spots segmented by the censored Gaussian mixture model (top panel) or the regular Gaussian mixture model (bottom panel). Foreground pixels are bounded by black line segments. Intermediate pixels that are neither foreground nor background are bounded between black and white line segments.

We use three spots with varying degrees of saturation to illustrate censored GMM-based clustering of pixels. Figure 3 displays the three spots, superimposed with model-based segmentation results by the censored GMM that corrects for saturated pixels or by the regular GMM without saturation adjustments. The pixel intensity values of the raw images were log transformed for a better contrast. Based on BIC, three-component mixture models were chosen for spots 1 and 2 in both methods and for spot 3 in the censored GMM. A two-component mixture was selected for spot 3 instead in the regular GMM. Spot 1 in Figure 3(a) and 3(b) has a circular shape and contains 34 saturated pixels. Spot 2 in Figure 3(c) and 3(d) appears to have an inner hole with 60 saturated pixels. Spot 3 depicted in Figure 3(e) and 3(f) is located on the upper edge of a block. It has 18 saturated pixels and manifests the so-called peak shape, that is, high intensity pixels are concentrated at the center while pixels with lower intensities fill up the rest of the "circle". From the figure, both model-based methods identified the circular spot (spot 1) and the donut-shaped spot (spot 2). For spot 3, the regular GMM failed to capture the peak shape due to treating saturated values as truly observed, whereas the censored GMM was able to separate the bright signal from its surrounding. The modified clustering results in the censored GMM demonstrate the need for saturation correction at the segmentation stage.

Table 1 reports FG median and mean intensities for the three spots from GenePix, the censored GMM, and the regular GMM. In GenePix, the size of a circular spot was fixed at 120. As the number of saturated pixels increases, the spot intensity estimated by GenePix also increases, but never exceeds *S*. The two model-based methods allow flexible spot shapes and varying spot sizes. Not accounting for saturation, the regular GMM cannot produce spot expression levels beyond *S*. In the extreme case of spot 2, the regular GMM grouped all the saturated pixels as the signal (i.e., the third component was a degenerate point mass at *S*), so the spot intensity is equal to *S*. In contrast, the censored GMM extends the dynamic range of expression levels, giving rise to spot intensity measures above *S *for spots 2 and 3 that have high percentages of saturated FG pixels (60*/*78 = 77% and 18*/*26 = 69%, respectively). About 29% of the FG pixels for spot 1 are saturated. In this case, the censored GMM reduces the downward bias with a larger spot intensity estimate than GenePix and the regular GMM.

**Table 1:** Foreground median and mean intensities for three saturated spots

Spot	Saturated pixels	FG pixels			FG median (mean)		
			
		GenePix	CGMM	GMM	GenePix	CGMM	GMM
1	34	120	116	114	52548 (48565)	57174	55538
2	60	120	78	60	59077 (42119)	70460	65535
3	18	120	26	140	20607 (26909)	74128	24738

Valley Fever diagnosis study: Number of saturated pixels, number of foreground pixels, and foreground median and mean intensities from GenePix, censored Gaussian mixture model, and regular Gaussian mixture model for the three saturated spots displayed in Figure 3

The BG intensities for the three spots are summarized in Table 2. The two sets of model-based estimates are close to each other and do not seem to be affected by saturated pixels. As a result, saturation adjustments in estimating the FG intensities are unlikely to be offset when BG-subtracted spot intensities are calculated. The GenePix BG intensity estimates are based on a much larger set of BG pixels for each spot and appear to be lower than the model-based estimates.

**Table 2:** Background median and mean intensities for three saturated spots

Spot	BG pixels			BG median (mean)		
		
	GenePix	CGMM	GMM	GenePix	CGMM	GMM
1	556	249	250	5784 (5825)	5845	5857
2	596	284	288	1619 (1992)	2458	2546
3	671	348	349	1038 (1107)	1243	1248

Valley Fever diagnosis study: Number of background pixels and background median and mean intensities from GenePix, censored Gaussian mixture model, and regular Gaussian mixture model for the three saturated spots displayed in Figure 3

### A canine lymphoma diagnosis study

Peptide microarrays from a canine lymphoma diagnosis study are used to illustrate the impact of saturation correction on downstream class prediction. The original images, obtained by scanning the arrays at 100% laser power and 70% PMT voltage, contained few saturated pixels, because most lymphoma tumors do not elicit a strong antibody response or the response is immunologically repressed. For the purpose of illustration, we censored the original pixel intensity values at artificial saturation thresholds to generate new data sets with desired saturation rates. An additional benefit of artificial censoring was to set a gold standard for comparing classification results, since in this case we know the original, uncensored data. In the analysis we included samples from 14 affected dogs and 7 healthy dogs. Each array consisted of a top sub-array and a bottom sub-array. A sub-array had 16 blocks, each containing 65 by 10 spots with around 400 pixels per target mask.

We applied artificial saturation thresholds *S*_1 _= 1000 and *S*_2 _= 800 to pixel intensity values on the 21 arrays and selected four spots as candidate peptides to be used for classifying a sample as healthy or with cancer. Our goal here was not to identify the complete set of peptides that contributed to class prediction. Instead, we used a small set of spots to demonstrate the potential impact of signal saturation on classification. Given the chosen saturation thresholds, the median percentages of saturated FG pixels across arrays in the new data sets were ranged from low to medium for *S*_1 _and from medium to high for *S*_2 _(see Table 3).

**Table 3:** Median percentage of saturated foreground pixels

Spot	Median % of saturated FG pixels	
	
	*S*_1 _= 1000	*S*_2 _= 800
1	3.3	27.9
2	4.9	50.0
3	10.9	69.4
4	26.5	72.2

Lymphoma diagnosis study: Median percentage of saturated foreground pixels, calculated as # of saturated foreground pixels divided by # of foreground pixels, across 21 arrays for four spots. The artificial saturation threshold was taken at *S*_1 _= 1000 or *S*_2 _= 800.

**Figure 4:**
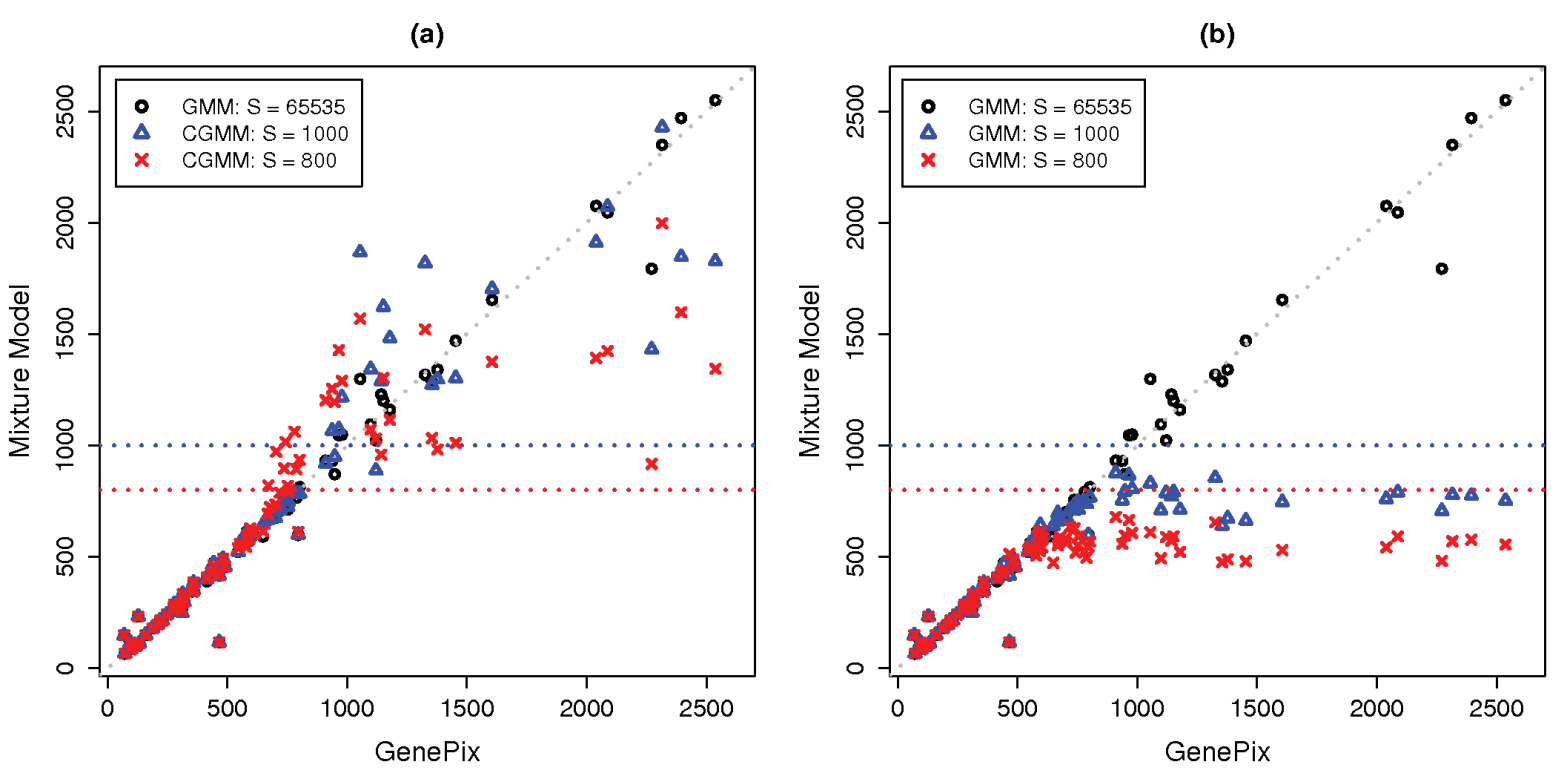
**Comparison of background-subtracted median intensities for four selected spots**. Lymphoma diagnosis study: Comparison of background-subtracted median intensity estimates for four spots on 21 arrays, based on the regular Gaussian mixture model and GenePix each with the original, uncensored data (*S *= 65535) as well as the censored Gaussian mixture model and the regular Gaussian mixture model each with the artificially saturated data (*S *= 1000 or 800).

Figure 4 compares BG-subtracted median intensity estimates for the four spots. First, we fit the original, uncensored data using both the regular GMM and GenePix. Figure 4 shows that the two sets of spot median intensities are in good agreement (i.e., black circles are close to the diagonal line). Next, the censored GMM and regular GMM were used to cluster the artificially censored data sets. Figure 4(a) suggests that the censored GMM is capable of correcting the downward bias. When the spot intensities are way above the saturation threshold (see the upper right corner of the plot), the adjustments tend to downshoot somewhat because a great amount of information on the signal is lost. In contrast, intensity estimates based on the regular GMM are severely biased downwards; see Figure 4(b). Three data points with median intensities ranged between 4000 and 8000 are not displayed for a better visualization of the rest of the data, but the results are similar.

Using BG-corrected median intensities for the four selected spots, each of the 21 samples was classified as healthy or having cancer based on logistic regression. The misclassification rates using leave-one-out cross validation are summarized in Table 4. The censored GMM has lower error rates at both saturation thresholds than the regular GMM. The higher the amount of saturated pixels (i.e., the smaller the saturation threshold), the larger the misclassification rate due to increased loss of information. Thus, correcting for signal saturation can potentially improve diagnostic accuracy.

**Table 4:** Misclassification rate based on leave-one-out cross validation

Method	Data	TP	TN	FP	FN	Error rate
GenePix	Original	12	4	3	2	0.24
GMM	Original	13	3	4	1	0.24
CGMM	Censored at 1000	13	3	4	1	0.24
GMM	Censored at 1000	10	2	5	4	0.43
CGMM	Censored at 800	11	3	4	3	0.33
GMM	Censored at 800	9	0	7	5	0.57

Lymphoma diagnosis study: Numbers of true positives (TP), true negatives (TN), false positives (FP) and false negatives (FN) out of 21 samples, and misclassification rate based on leave-one-out cross validation

### Simulation

A simulation study was performed to investigate the use of information criteria on mixture component selection and the performance of our optimization algorithm implementation. A *K*-component GMM, *K *= 2 or 3 with one component right-censored at *S *= 65, 535, was used as the true model. At each *K*, the parameters were chosen to mimic the Valley Fever diagnosis study and allow 10%, 40% or 70% of saturated FG pixels. The number of pixels within a target mask was fixed at 500. 1000 simulated trials were run in R for each of the six settings. Each simulated data set was fitted by both the censored GMM and the regular GMM. The complete, uncensored data were also analyzed by the regular GMM. Akaike information criterion (AIC) and BIC were used to select *K *in each model.

The EM algorithm converged fast in all simulation runs. In addition, we observed that spot intensity estimates were somewhat robust to the choice of EM initial values. Table 5 summarizes the percent of times *K *was correctly selected by BIC; the results based on AIC are similar and not shown. In the regular GMM for fitting uncensored data, the correct number of mixture components was chosen for all simulated runs, regardless of the true *K*. The censored GMM with censored data correctly selected *K *in all except for five trials (when the true *K *was three and there were 70% of saturated FG pixels). When the percentage of saturation was small, the regular GMM with censored data identified *K *correctly most of the time. With medium and high amounts of saturated FG pixels, however, the chance of choosing an incorrect *K *increases. For example, when fitting the censored data generated under *K *= 3 and 70% of saturation, a three-component regular GMM was selected only 69% of times; for the rest trials a two-component GMM was selected as the final model based on BIC, causing severe downward bias in estimating spot intensities. This provides additional evidence that treating saturated values as truly observed, as in a regular GMM, can alter the selection on the number of clusters and result in modified cluster memberships. Thus, accounting for signal saturation at the image segmentation stage may lead to more accurate spot intensity estimation.

**Table 5:** Percent of times the mixture component was correctly selected

*K*	% of saturated FG pixels	GMM0	CGMM	GMM1
2	10	100.0	100.0	99.0
	40	100.0	100.0	76.3
	70	100.0	100.0	82.7
3	10	100.0	100.0	100.0
	40	100.0	100.0	95.0
	70	100.0	99.5	68.9

Percent of times the mixture component *K *was correctly selected by BIC in the regular Gaussian mixture model for complete, uncensored data (GMM0), the censored Gaussian mixture model for censored data, and the regular Gaussian mixture model for censored data (GMM1)

In Table 6 and Table 7, we report the simulation behavior of parameter estimates. Only the simulated trials in which *K *was correctly chosen were included to separate the effect of an incorrect number of clusters from the impact of signal saturation given that *K *was correctly identified. The relative bias for a parameter is defined as the difference between the mean simulation parameter estimate and the true parameter value, divided by the true value. The tables suggest that, with moderate to high amounts of saturated FG pixels, the regular GMM-based spot intensity estimates are seriously biased downwards. In contrast, the censored GMM extends the dynamic range of the signal and effectively corrects the negative bias in estimating the FG signal over a wide range of saturation percentages. Estimation of BG intensities is not affected by whether or not saturation is accounted for.

**Table 6:** Relative bias in the two-component mixture model

Parameter	GMM0			CGMM			GMM1		
			
	10	40	70	10	40	70	10	40	70
*π*_1 _= 0.8	0.0006	0.0007	0.0006	0.0006	0.0006	0.0006	0.0026	0.0075	0.0035
*μ*_1 _= 8, 000	0.0003	0.0003	0.0003	0.0003	0.0003	0.0003	0.0007	0.0006	0.0014
*σ*_1 _= 2, 000	-0.0043	-0.0043	-0.0044	-0.0044	-0.0044	-0.0045	-0.0010	0.0032	0.0073
*μ*_2_	-0.0002	0.0000	-0.0001	-0.0001	0.0004	-0.0019	-0.0104	-0.0867	-0.2220
*σ*_2_	-0.0079	-0.0080	-0.0075	-0.0070	-0.0068	-0.0162	-0.1121	-0.3784	-0.6426

Simulation with true *K *= 2: Relative bias based on runs with *K *correctly selected by BIC. The models considered were regular Gaussian mixture for complete, uncensored data (GMM0), censored Gaussian mixture for censored data, and regular Gaussian mixture for censored data (GMM1). Percents of saturated foreground pixels were set at 10% (*μ*_2 _= 46, 300, *σ*_2 _= 15, 000), 40% (*μ*_2 _= 60, 450, *σ*_2 _= 20, 000) and 70% (*μ*_2 _= 78, 650, *σ*_2 _= 25, 000).

**Table 7:** Relative bias in the three-component mixture model

Parameter	GMM0			CGMM			GMM1		
			
	10	40	70	10	40	70	10	40	70
*π*_1 _= 0.7	0.0011	0.0010	0.0011	0.0011	0.0011	0.0012	0.0006	-0.0002	-0.0088
*μ*_1 _= 2, 000	0.0015	0.0015	0.0015	0.0015	0.0015	0.0015	0.0015	0.0014	0.0033
*σ*_1 _= 1, 000	-0.0044	-0.0045	-0.0045	-0.0044	-0.0045	-0.0044	-0.0051	-0.0077	-0.0117
*π*_2 _= 0.1	-0.0022	-0.0017	-0.0023	-0.0032	-0.0032	-0.0046	0.0176	0.1637	0.5294
*μ*_2 _= 15, 000	0.0008	0.0005	0.0004	0.0003	-0.0001	0.0003	0.0117	0.1679	0.7195
*σ*_2 _= 6, 000	-0.0262	-0.0249	-0.0255	-0.0276	-0.0271	-0.0285	0.0100	0.3923	1.8881
*μ*_3_	-0.0001	0.0000	-0.0002	-0.0002	0.0002	-0.0010	-0.0053	-0.0384	-0.1352
*σ*_3_	-0.0071	-0.0076	-0.0071	-0.0049	-0.0028	-0.0033	-0.1161	-0.4601	-0.9294

Simulation with true *K *= 3: Relative bias based on runs with *K *correctly selected by BIC. The models considered were regular Gaussian mixture for complete, uncensored data (GMM0), censored Gaussian mixture for censored data, and regular Gaussian mixture for censored data (GMM1). Percents of saturated foreground pixels were set at 10% (*μ*_3 _= 52, 700, *σ*_3 _= 10, 000), 40% (*μ*_3 _= 62, 500, *σ*_3 _= 12, 000) and 70% (*μ*_3 _= 75, 000, *σ*_3 _= 18, 000).

## Conclusions

In analysis of expression microarrays, the issue of signal saturation has been frequently neglected, causing downward bias in estimating spot expression levels and potentially distorting high-level analysis. Previous work has focused on saturation correction based on already segmented data, at either the spot or pixel level. In this article, we combine model-based segmentation and spot intensity estimation into an integrated procedure that has the potential to recover or partially recover the lost information on expression levels due to saturated pixels. The procedure models saturated pixels as right censored at the saturation threshold and is implemented in R for high-throughput analysis. As demonstrated in microarray examples and simulation, the proposed method extends the dynamic range of expression data at the high end, is effective in correcting saturation-induced bias at the pixel level, better identifies the cluster memberships of pixels, and has the potential to increase the predictive power for downstream class prediction. As a model-based segmentation method, our procedure can identify inner holes, fuzzy edges and blank spots that are common in microarray images. Although illustrated with single-dye peptide microarrays, the approach is independent of microarray platform and applicable to both single- and dual-channel microarrays. Our method does not need multiple scans of an image to achieve saturation correction as required by some of the existing methods.

Possible extensions to the current work include development of model-based image segmentation procedures that assume robust or other distributions for the foreground and background pixels [8,15] and also account for signal saturation. The spatial information in a microarray image should also be exploited in different means to more effectively guide the model-based clustering of pixels.

## Authors' contributions

YY was responsible for model construction, implementation of the methods, data analysis, and paper writing. PS provided the data, validated the results, and helped with downstream classification and paper writing. YK helped with implementation. All authors have read and approved the final manuscript.
